# Development and evaluation of fluorescent recombinase polymerase amplification (RPA)-based method for rapid detection of *Necator americanus*

**DOI:** 10.1371/journal.pntd.0013007

**Published:** 2025-04-08

**Authors:** Jia-Rui Liang, Shu-Ning Yan, Han-Yin Yang, Shuo Yang, Yu-Juan Shen, Le-Le Huo, Yu-Chun Cai, Zi-Ran Mo, Bin Zheng, Bin Xu, Wei Hu

**Affiliations:** 1 National Key Laboratory of Intelligent Tracking and Forecasting for Infectious Diseases, National Institute of Parasitic Diseases at Chinese Center for Disease Control and Prevention, Chinese Center for Tropical Diseases Research, Shanghai, China; 2 National Institute of Parasitic Diseases, Chinese Center for Disease Control and Prevention (Chinese Center for Tropical Diseases Research), National Health Commission Key Laboratory of Parasite and Vector Biology, WHO Collaborating Center for Tropical Diseases, National Center for International Research on Tropical Diseases, Shanghai, China; 3 The institutes of Biomedical Sciences, College of Life Sciences, Inner Mongolia University, Hohhot, Inner Mongolia, China; 4 School of Global Health, Chinese Center for Tropical Diseases Research, Shanghai Jiao Tong University School of Medicine, Shanghai, China; 5 Department of Microbiology and Microbial Engineering, State Key Laboratory of Genetic Engineering, Ministry of Education Key Laboratory of Contemporary Anthropology, School of Life Sciences, Fudan University, Shanghai, China; NEHU: North Eastern Hill University, INDIA

## Abstract

**Background:**

*Necator americanus* is the predominant species causing hookworm infections in humans. Despite advancements in prevention strategies, mild cases of infection still occur, highlighting the need for improved detection technology. Recombinase Polymerase Amplification (RPA) is an isothermal molecular diagnostic known for its sensitivity, speed, portability, and widespread application in detecting various pathogens. Although several molecular assays are available for *N. americanus*, they have limitations in detecting mild *N. americanus* infections.

**Methods:**

Fluorescent RPA primers and probes targeting the *N. americanus* internal transcribed spacer 2 (*ITS2)* gene were developed. The method’s detection limit was assessed via serial dilution of genomic DNA. Specificity was confirmed against *Clonorchis sinensis, Schistosoma japonicum, Fasciola hepatica, Ascaris lumbricoides, Enterobius vermicularis* and *Ancylostoma duodenale*. Thirty samples identified as positive by Kato-Katz, along with 11 samples identified as negative by the method, were tested to evaluate the sensitivity and specificity of fluorescent RPA. Additionally, 287 field samples were tested for validation with these methods. All positive samples were identified as either *N. americanus* or *A. duodenale*.

**Results:**

This study successfully developed a fluorescent RPA assay targeting the *ITS2* gene of *N. americanus*. The length of the amplified fragment was 237 bp. Optimized conditions were achieved, resulting in a minimum detection limit of 1fg/µL, with no cross-reactivity with other pathogens. In laboratory validation, the fluorescent RPA assay demonstrated 100% sensitivity (30/30) and 100% specificity (11/11) compared to the Kato-Katz, and 100% sensitivity (29/29) and 91.7% specificity (11/12) when compared to the semi-nested PCR. In field validation using human fecal samples, the fluorescent RPA assay showed a sensitivity of 90.0% (36/40) and a specificity of 91.1% (225/247) compared to the Kato-Katz. And the sensitivity of the fluorescent RPA method compared to the semi-nested PCR method was 100% (34/34), while the specificity was 90.5% (229/252).

**Conclusions:**

The fluorescent RPA assay presents a rapid and dependable method for detecting *N. americanus* in fecal samples. Its high sensitivity and specificity provide significant utility for field surveillance and early identification of *N. americanus* infections. This advancement could facilitate the rapid molecular diagnosis of *N. americanus* disease in hookworm-endemic regions.

## Author summary

The fluorescent recombinase polymerase amplification (RPA) technique is a state-of-the-art method for isothermal DNA amplification, utilizing a portable instrument and lyophilized reagents. This innovative approach allows for on-site testing using a portable fluorescence detector, significantly enhancing its utility in field settings. In our study, we evaluated 287 samples obtained directly from the field. The conventional semi-nested PCR assay demonstrated a sensitivity of 62.5% and a specificity of 96.4%. In contrast, the newly developed fluorescent RPA assay exhibited superior performance, with a sensitivity of 90.0% and a specificity of 91.1% for detecting *N. americanus* infection. These findings highlight the potential of fluorescent RPA as a more sensitive method for identifying *N. americanus*. Moreover, our research suggests that the established fluorescent RPA could play a crucial role in various applications, such as surveillance and epidemiological and clinical investigations related to *N. americanus*. Further studies are warranted to explore its full potential and optimize its implementation in practical settings. This advancement represents a significant step forward in the field of diagnostic techniques for parasitic infections.

## 1. Introduction

*Necator americanus*, a parasitic nematode found in the human small intestine, is one of the primary hookworm species, along with *Ancylostoma* spp [1]. This species is predominant and exhibits a particularly high prevalence in regions including southern China, Southeast Asia, the Americas, and much of Africa [2]. Adult worms reproduce and lay eggs in the duodenum, while their larvae can penetrate human skin, undergo several molts, and eventually mature into adult worms, a process that may lead to disease development, such as blood-feeding in the gastrointestinal tract, causing hemorrhaging and contributing to iron deficiency anemia (IDA) [2,3]. The impact of *N. americanus* on human health is significant, especially in areas with poor sanitation, where the transmission cycle is more easily sustained [1]. Addressing this parasitic infection requires a thorough understanding of the hookworm’s biology and behavior, along with advancements in diagnostic methods to enable early detection and mitigate its impact on affected populations.

Understanding the unique characteristics of hookworm species, particularly *N. americanus*, emphasizes the need for precise detection methods with high sensitivity and specificity to develop effective control strategies [2,4–6]. Individuals with *N. americanus* often have low worm burdens, making conventional diagnostic assays prone to under-detection [7–9]. Additionally, the co-endemicity of *N. americanus* with *Ancylostoma* spp in certain regions complicates diagnostic specificity, potentially leading to misclassification or overlooking specific hookworm species [10–12]. These factors highlight the importance of improving assay sensitivity and specificity to reduce the risk of misdiagnosis.

Recent advancements in molecular diagnostics, such as quantitative real-time multiplex PCR and isothermal loop amplification (LAMP), have significantly enhanced the detection of parasitic infections, exemplified by studies identifying intestinal parasites in various populations and addressing the limitations of conventional methods [13–15]. Recombinase Polymerase Amplification (RPA) is an isothermal DNA amplification technique that utilizes portable instrumentation and lyophilized reagents, enabling rapid reactions at 37–42°C and minimal resource requirements [16]. Detection of amplified DNA is feasible via gel electrophoresis, fluorescence analysis, or oligochromatographic lateral flow (LF) strips, making RPA technology an ideal choice for field deployment [17–19]. Fluorescent RPA with probe technology enhances sensitivity and specificity compared to conventional PCR [20,21]. Its compatibility with portable devices (e.g., Genie III, Twista) makes it well-suited for rapid on-site testing in low-resource settings [22–26].

This study aims to establish a rapid detection method for detecting *N. americanus* in fecal samples using the fluorescent RPA. This endeavor seeks to provide technical support for the monitoring and early warning of *N. americanus* infections.

## 2. Methods

### 2.1. Ethics statement

The animal experiments were approved by the Animal Welfare and Ethics Committee of the National Institute of Parasitic Diseases, Chinese Center for Disease Control and Prevention (Permit No: IPD-2019–6).

This study was approved by the Medical Ethics Committee of the Institute of Parasitic Disease Prevention and Control of the Chinese Center for Disease Control and Prevention (National Tropical Disease Research Center) (ethical approval number 2021019). Samples were collected after obtaining the informed verbal consent from the subjects or their families.

### 2.2. Sample collection

#### 2.2.1. Parasites.

The Institute of Parasitic Disease Prevention and Control of the Chinese Center for Disease Control and Prevention (National Center for Tropical Disease Research) provided the larvae of *Clonorchis sinensis, Schistosoma japonicum, Fasciola hepatica, Ascaris lumbricoides, Enterobius vermicularis and Ancylostoma duodenale*. Hamsters’ fecal samples were collected from an established animal model of hamsters infected with *N. americanus*, and the mice were obtained from the same institution.

#### 2.2.2. Human fecal sample collection.

Forty-one human fecal samples, collected from Zhejiang and Hainan Provinces between 2020 and 2021, were tested using Kato-Katz conducted immediately after collection, stored in 75% ethanol at 4°C for nucleic acid extraction, and later used to evaluate and compare the sensitivity and specificity of the fluorescent RPA. Laboratory staff provided 40 healthy human fecal samples, each one was artificially spiked with either 1 or 3 eggs of *N. americanus*. In addition, a total of 287 human fecal samples were collected from field sites in Hainan and Zhejiang Provinces in 2022 for examination. These included 273 samples from Baoting Li and Miao Autonomous County, Changjiang Li Autonomous County, and Tanniu Town of Wenchang City in Hainan Province, along with 14 samples from Linhai City and Ninghai County in Zhejiang Province. Following microscopic examination using the Kato-Katz, all human fecal samples were immersed in 75% alcohol and stored at 4°C for nucleic acid extraction.

### 2.3. Preparation of human fecal samples with different *N. americanus* egg density

To assess the feasibility of the developed method for detecting samples with low infection intensity, *N. americanus* eggs were isolated from the feces of infected hamsters through flotation in saturated saline. The clarified supernatants containing *N. americanus* eggs were pooled, and egg counts were conducted under low magnification, yielding a concentration of 5 eggs per 10 µL. Low concentrations of *N. americanus* eggs (i.e., 5 or 15 eggs per gram [EPG]) were then spiked into 200 mg aliquots of human STH-negative feces, and the procedure was repeated 20 times. Following this, all prepared fecal samples were immersed in 75% alcohol and stored at 4°C for nucleic acid extraction.

### 2.4. DNA extraction

Genomic DNA were directly extracted from the larvae of the parasite as *C. sinensis*, *S. japonicum, F. hepatica*, *A. lumbricoides*, *E. vermicularis*, *N. americanus* and *A. duodenale* larvae using the Tissue and Blood Kit (QIAGEN, Germany), with 25 mg of tissue and 200 µL of elution buffer. Forty fecal samples containing varying numbers of *N. americanus* eggs prepared in the laboratory, alongside 41 samples for laboratory validation and 287 samples for field validation, were thoroughly mixed, and 200 mg samples were then taken using a three-point sampling method from three distinct locations: the surface, center, and periphery, to ensure that different areas of the feces were well-represented. Samples were processed using a Bertin Precellys 24 (Bertin Technologies, France) with centrifuge tubes containing steel beads to mechanically disrupt the fragile eggshell layer. The samples were homogenized at 6500 rpm for 20 seconds, repeated twice, for a total of three cycles. The subsequent steps followed the QIAamp Fast DNA Stool Mini Kit (QIAGEN, Germany) protocol, utilizing 200 mg of stool and 200 µL of elution buffer. Extracted DNA was stored at -20°C for further analysis., facilitating genomic DNA extraction. The subsequent steps followed the QIAamp Fast DNA Stool Mini Kit (QIAGEN, Germany) protocol, utilizing 200 mg of stool and 200 µL of elution buffer. Extracted DNA was stored at -20°C for further analysis.

### 2.5. The fluorescent RPA assay

#### 2.5.1. Design and screen of primers and probes.

**Fig 1:**
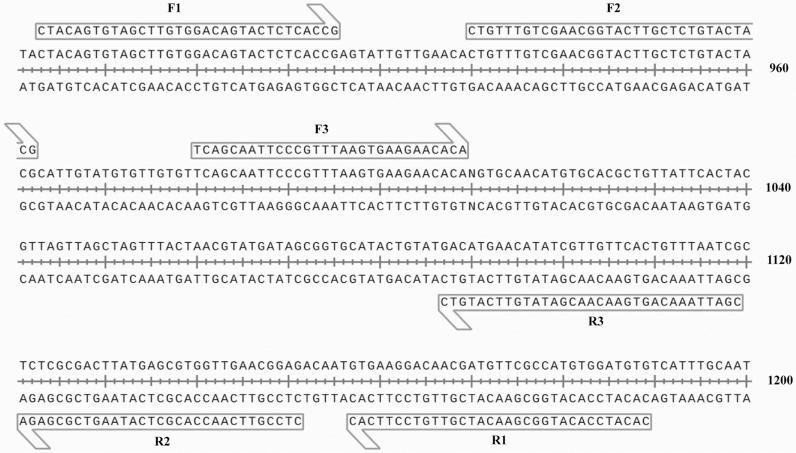
The positions of the primers designed in this study.

In this study, the *ITS2* region (GenBank accession no. LC036565.1) of *N. americanus* was chosen as the target gene. According to the manuals of the TwistAmp exo kits (TwistDx, Cambridge, UK), fluorescent RPA oligonucleotides were custom-tailored to target the *N. americanus* gene segment. Due to the extended length of fluorescent RPA primers and probes, it was imperative to mitigate non-specific amplification, such as primer-primer and primer-probe interactions. For this reason, primer pairs were initially aligned and combined, resulting in a total of 9 primer combinations selected through a random combination approach. Subsequently, a negative control was performed using sterile water to exclude primer pairs that yielded non-specific fluorescence signals. Primer pairs devoid of non-specific fluorescence amplification advanced to subsequent rounds of screening. The optimal primer pair targeting the specified sequence was determined through fluorescence amplification using *N. americanus* genomic DNA as a template. Fluorescence signal reactions and readings were conducted using CFX96 fluorescence quantitative PCR instrument (Bio-Rad, the USA). Selection criteria were based on the intensity of Relative Fluorescence Units (RFU) and the number of cycles in which fluorescence occurs. The details of these primer pairs, along with the fluorescent probe (P), are summarized in Table 1. The positions of the six primers designed for the fluorescent RPA are shown in Fig 1.

**Table 1:** Primers and probe sequences for the *ITS2* of *N. americanus and A. duodenale.*

Primers and probe	Methodology	Sequence	Reference
F1	Fluorescent RPA	CTACAGTGTAGCTTGTGGACAGTACTCTCACCG	In this study
F2	Fluorescent RPA	CTGTTTGTCGAACGGTACTTGCTCTGTACTACG	In this study
F3	Fluorescent RPA	TCAGCAATTCCCGTTTAAGTGAAGAACACA	In this study
R1	Fluorescent RPA	CACATCCACATGGCGAACATCGTTGTCCTTCAC	In this study
R2	Fluorescent RPA	CTCCGTTCAACCACGCTCATAAGTCGCGAGA	In this study
R3	Fluorescent RPA	CGATTAAACAGTGAACAACGATATGTTCATGTC	In this study
P	Fluorescent RPA	CTGTTATTCACTACGTTAGTTRGCTAGTT/i6FAMdT//idSp/C/iBHQdT/AACGTATGATAGCG-C3 Spacer	In this study
NC1	Semi-nested PCR	ACGTCTGGTTCAGGGTTGTT	[27–29]
NC2	Semi-nested PCR	TTAGTTTCTTTTCCTCCGCT	[27–29]
NA	Semi-nested PCR	ATGTGCACGTTATTCACT	[27–29]
AD	Semi-nested PCR	CGACTTTAGAACGTTGC	[29]

#### 2.5.2. RPA reaction system.

RPA reactions were performed using the TwistAmp exo kit (TwistDx, Cambridge, UK) in 50 µL of reaction systems, utilizing CFX96 fluorescence quantitative PCR instrument (Bio-Rad, the USA). Each reaction contained 25 µL of buffer, 2.1 µL of each forward and reverse primer (10 µM), 0.6 µL of probe (10 µM), 2 µL of template genomic DNA, and 15.2 µL of distilled water (ddH_2_O), which were added to the lyophilized RPA pellet. Subsequently, 3 µL of magnesium acetate (280 mmol/L) was added to the lid of the pellet. The reaction was initiated by centrifugation of the pellet, followed by reaction of the tubes at 39°C for 20 minutes.

#### 2.5.3. Sensitivity and specificity of RPA assays.

To assess the sensitivity and specificity of the RPA assays with optimal primers and probe. Genomic DNA from *N. americanus* worms were serially diluted to concentrations of 100 pg/µL, 10 pg/µL, 1 pg/µL, 100 fg/µL, 10 fg/µL, 1 fg/µL, and 0.1 fg/µL to serve as amplification templates. The experiment was conducted in triplicate. Fluorescent RPA was then conducted using the optimal primer pairs identified to determine the lowest detection limit of the method. Furthermore, 40 artificially prepared fecal samples (including 20 samples containing a single *N. americanus* egg and 20 samples containing three *N. americanus* eggs, i.e., 5 or 15 eggs per gram [EPG]) were further tested for sensitivity using semi-nested PCR and fluorescent RPA.

Additionally, genomic DNA samples from *C. sinensis, S. japonicum, F. hepatica, A. lumbricoides*, *E. vermicularis* and *A. duodenale* were utilized as templates for fluorescent RPA to assess the presence of cross-reactivity.

A genomic DNA template of *N. americanus* at a concentration of 10 fg/µL was employed as the positive control. Distilled water was employed as the negative control. A no template control (NTC) is equivalent to blank control. The shape of the amplification curves was used to assess whether a sample is positive or negative. Specifically, if the amplification curve follows the ‘S’-shaped trend, it is determined as positive [24]. Conversely, if the trend resembles that of the negative control, it is judged as negative. The experiments were conducted in triplicate to ensure the reliability and reproducibility of the results.

### 2.6. Kato-Katz

The Kato-Katz was employed as the “gold standard” to evaluate and compare the sensitivity and specificity of both the semi-nested PCR and fluorescent RPA. All samples underwent Kato-Katz testing. The Kato-Katz assay was conducted according to the manufacturer’s instructions using a detection kit (Yiman Biotechnology Co., Ltd, China). This involved preparing Kato tablets with nylon gauze, scrapings, slides, and so on, followed by microscopic examination of the smears under 10x magnification and double fecal testing to detect *N. americanus* eggs. The slides were processed in batches, with the interval between preparation and examination not exceeding one hour. If eggs were detected, the result was considered positive, and the average number of eggs in two Kato-Katz discs was calculated. If no eggs were detected, the result was considered negative.

### 2.7. Semi-nested PCR

To benchmark against the established fluorescent RPA, semi-nested PCR was chosen for the assay. All samples were subjected to semi-nested PCR. The semi-nested PCR primers were synthesized based on the literature [27] and the primer sequences are listed in Table 1. The reaction was performed in a total volume of 25 µL solution consisting of 12.5 µL of 2×PCR mix (TAKARA, 320A, Japan), 1 µL of forward primer (10 µM), 1 µL of reverse primer (10 µM), 1 µL of extracted DNA from fecal samples, and 9.5 µL of ddH_2_O. All PCR reactions were conducted using T100 Thermal Cycler (Bio-Rad, the USA). For *N. americanus* and *A. duodenale*, the reaction conditions were identical, with both utilizing NC1 and NC2 as the forward and reverse primers, respectively, and the protocol of the first reaction round was as follows: initial denaturation at 94°C for 5 minutes, followed by 25 cycles consisting of denaturation at 94°C for 30 seconds, annealing at 55°C for 30 seconds, and extension at 72°C for 30 seconds, concluding with a final extension step at 72°C for 5 minutes [27–29]. The second round of PCR reactions for *N. americanus* was carried out using NA and NC2 as forward and reverse primers, respectively [27,28], the product length of nested PCR was 230 bp. The reaction procedure was as follows: initial denaturation at 94°C for 5 minutes, followed by 35 cycles consisting of denaturation at 94°C for 30 seconds, annealing at 55°C for 30 seconds, and extension at 72°C for 30 seconds, concluding with a final extension step at 72°C for 5 minutes. As for *A. duodenale*, the second round of PCR reactions employed AD and NC2 as forward and reverse primers, the length of the amplified fragment is 130 bp. The cycling was following conditions: initial denaturation at 94°C for 5 minutes, followed by 35 cycles consisting of denaturation at 94°C for 60 seconds, annealing at 55°C for 60 seconds, and extension at 72°C for 60 seconds, concluding with a final extension step at 72°C for 5 minutes [29] The PCR products were verified by 2% agarose gel electrophoresis and Sanger sequencing (Beijing Liuhe Huada Gene Technology Co). The sequences were compared with similar sequences retrieved from the GenBank database using BLASTn (http://www.ncbi.nlm.nih.gov/BLAST/).

### 2.8. Data statistical analysis

A total of 41 human fecal samples were analyzed using Kato-Katz, semi-nested PCR, and the established fluorescent RPA for sensitivity and specificity validation, while 287 field samples were tested using the same methods. Fisher’s exact probability test, along with the calculation of detection specificity, sensitivity, PPV, and NPV, was performed using SAS 9.4.

## 3. Results

### 3.1. The primers and probe for fluorescent RPA

The *ITS2* upper and lower primers were aligned and paired, and ddH_2_O was utilized as negative control to eliminate primer combinations potentially causing non-specific fluorescence amplification. As shown in Table 1, 3 forward primers, 3 reverse primers and 1 probe were designed. Thus, there were 9 types of primer combinations, i.e., F1-R1 (1), F1-R2 (2), F1-R3 (3), F2-R1 (4), F2-R2 (5), F2-R3 (6), F3-R1 (7), F3-R2 (8), and F3-R3 (9).

**Fig 2:**
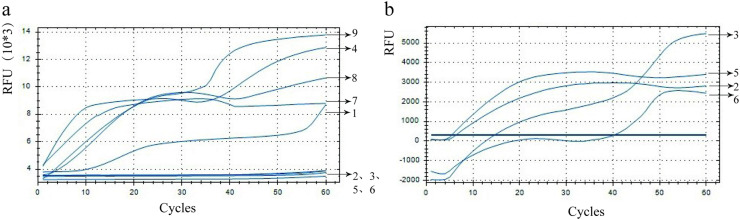
Results of primers and probe screening for *ITS2* gene. (a) Primer-probe interference experiments. (b) Optimization results of the primer combinations for detecting *N. americanus* eggs.

Fig 2a shows the interferences between these primer combinations and the probe. The x-axis represents cycles, while the y-axis represents relative fluorescence units (RFU). Briefly, primer combinations labelled 2, 3, 5, and 6 exhibited no fluorescent amplification in the presence of no templates, suggesting non-coupling of the primers with the probe. Thus, these four different primer combinations (2, 3, 5 and 6) were further used for fluorescent amplification of the genomic DNA of *N. americanus* eggs. As shown in Fig 2b, the four primer combinations exhibited similar peak times. Particularly, the primer combination No. 3 (F1-R3) showed the most significant change in fluorescence intensity and the amplification length of this primer pair was 237 bp. Therefore, this primer combination was selected as the final forward and reverse primer for this method (Fig 2).

### 3.2. Laboratory evaluation of the fluorescent RPA

#### 3.2.1. Analytical sensitivity and specificity of RPA assays.

**Fig 3:**
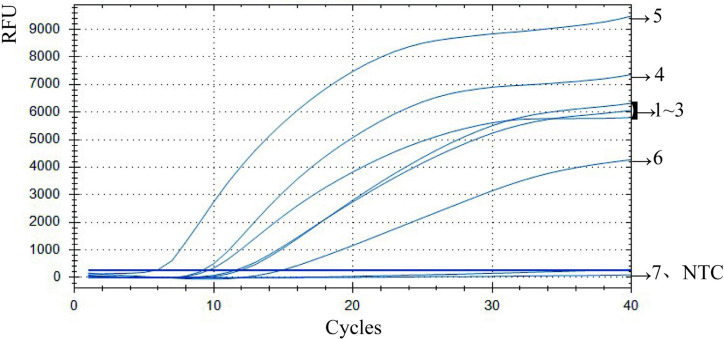
Determination of the minimum *ITS2* detection limit of the RPA assay using the optimal primer combination. Curves 1- 7 shows the amplification fluorescence curves of *N. americanus* genomic DNA samples with pre-set concentrations of 100 pg/µL, 10 pg/µL, 1 pg/µL, 100 fg/µL, 10 fg/µL, 1 fg/µL, and 0.1 fg/µL, respectively. NTC: No template control.

Using the optimal primer combination 3 (F1-R3), *N. americanus* genomic DNA samples with pre-set concentrations of 100 pg/µL, 10 pg/µL, 1 pg/µL, 100 fg/µL, 10 fg/µL, 1 fg/µL, or 0.1 fg/µL were detected via the RPA assay to assess the analytical sensitivity of this method. As shown in Fig 3, significant fluorescence curves were observed when the concentration of *N. americanus* genomic DNA samples were ≥1 fg/µL. Conversely, when the concentration of *N. americanus* genomic DNA samples were ≤0.1 fg/µL, the amplification curve resembled that of the NTC, with no detectable fluorescence amplification. Therefore, the method’s minimum detection limit was established as 1 fg/µL.

**Fig 4:**
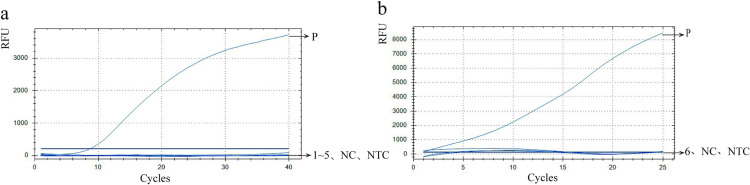
Cross reaction test of fluorescence RPA (a) Cross reaction test with *C. sinensis, S. japonicum, F. hepatica, A. lumbricoides*, *E. vermicularis* (Line 1-5: Genomic DNA of *C. sinensis, S. japonicum, F. hepatica, A. lumbricoides* and *E. vermicularis*; PC: Positive control; NC: Negative control; NTC: No template control) (b) Cross reaction test with *A. duodenale* (Line6: Genomic DNA of *A. duodenale*; PC: Positive control; NC: Negative control; NTC: No template control).

To further detect the specificity of the RPA assay, *N. americanus* genomic DNA samples as well as the genomic DNA samples of *C. sinensis, S. japonicum, F. hepatica, A. lumbricoides*, *E. vermicularis* and *A. duodenale* were further tested via the RPA assay with the same optimal primer combination. As shown in Fig 4, the positive control (*N. americanus* genomic DNA) successfully generated an ‘S’-shaped trend fluorescence curve, indicating amplification. Besides, the genomic DNA fluorescence amplification curves of *C. sinensis, S. japonicum, F. hepatica, A. lumbricoides*, *E. vermicularis* and *A. duodenale* overlapped with those of the negative control and the NTC, showing no fluorescence amplification. This observation indicates a high degree of specificity in the well-established fluorescent RPA response of *N. americanus*.

#### 3.2.2. Detection of fecal samples containing varying quantities of *N. americanus* eggs.

**Fig 5:**
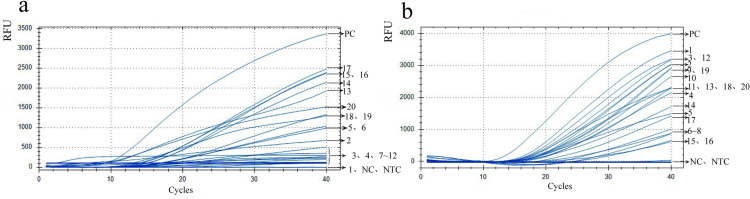
Detection results of fecal samples containing varying quantities of *N. americanus* eggs. (a) Detection results of fecal samples containing 1 egg by fluorescent RPA. (b) Detection results of fecal samples containing 3 eggs by fluorescent RPA.

DNA extracted from 40 artificially prepared fecal samples, including 20 samples containing one egg of *N. americanus* and 20 samples containing three eggs of *N. americanus*, and the positive control, negative control as well as NTC, were detected using both semi-nested PCR and fluorescent RPA. The genomic DNA fluorescence amplification curves of *N. americanus* in these samples detected via fluorescent RPA and semi-nested PCR are shown in Fig 5. Meanwhile, the detection data for the samples containing one egg and the samples containing 3 eggs are summarized in Tables 2 and 3, respectively. As shown in Table 2, Fig 5a and S1a Fig, out of the 20 samples containing one egg, 12 samples (detection rate: 60%) were identified as *N. americanus* positive and 8 samples were negative by semi-nested PCR, while 19 samples (detection rate: 95%) were detected as positive by fluorescent RPA. For samples containing three eggs, semi-nested PCR identified 19 samples as *N. americanus* positive and 1 sample as negative, resulting in a detection rate of 95% (19/20). Moreover, all 20 samples analyzed using fluorescent RPA displayed fluorescence amplification curves that met the positivity criteria, resulting in a detection rate of 100% (20/20) (Fig 5b, S1b Fig and Table 3).

**Table 2:** Detection results of semi-nested PCR and fluorescence RPA assay for 20 samples each containing one egg of *N. americanus.*

Fluorescence RPA	Semi-nested PCR		Total
	+	−	
+	12	7	19
−	0	1	1
Total	12	8	20

**Table 3:** Detection results of semi-nested PCR and fluorescence RPA assay for 20 samples each containing three egg of *N. americanus.*

Fluorescence RPA	Semi-nested PCR		Total
	+	−	
+	19	1	20
−	0	0	0
Total	19	1	20

#### 3.2.3. Detection of the 41 fecal samples with different methods.

**Fig 6:**
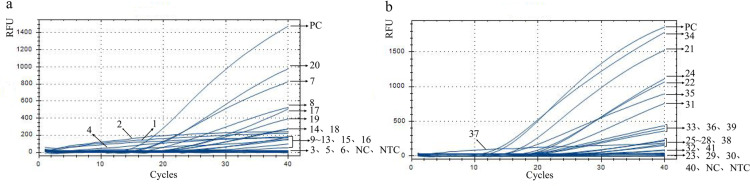
Detection results of *N. americanus* in 41 Human Fecal Samples via Fluorescent RPA method for Sensitivity and Specificity Assessment (Line 1-41: Human fecal samples; PC: Positive control; NC: Negative control; NTC: No template control).

A total of 41 samples, of which 30 were Kato-Katz positive and 11 were Kato-Katz negative, were tested using semi-nested PCR and fluorescence RPA to evaluate the sensitivity and specificity of fluorescence RPA. The raw data of detections are recorded in Table 4, the amplification curves are shown in Fig 6, and the analysis data are shown in Table 5. Table 4 shows the sample number, and the detection results of these samples via the three methods. Fig 6 and S2 Fig presents the results of the fluorescent RPA assay and semi-nested PCR.

**Table 4:** Raw data of detection results of 41 samples using Kato-Katz, semi-nested PCR and fluorescent RPA.

Samples	Kato-Katz (average egg number)	Semi-nested PCR	Fluorescence RPA
1	0	−	−
2	0	−	−
3	0	−	−
4	0	−	−
5	0	−	−
6	0	−	−
7	26	+	+
8	150	+	+
9	2.5	+	+
10	1	−	+
11	1	+	+
12	6	+	+
13	3	+	+
14	3	+	+
15	6.5	+	+
16	2	+	+
17	65	+	+
18	6	+	+
19	15	+	+
20	290	+	+
21	35	+	+
22	1	+	+
23	0	−	−
24	231	+	+
25	3	+	+
26	43	+	+
27	2	+	+
28	2	+	+
29	0	−	−
30	0	−	−
31	4	+	+
32	4	+	+
33	23	+	+
34	44	+	+
35	2	+	+
36	14	+	+
37	0	−	−
38	2	+	+
39	17	+	+
40	0	−	−
41	7	+	+

**Table 5:** Laboratory validation of the Kato-Katz for human fecal samples compared with fluorescent RPA and semi-nested PCR assays.

Kato-Katz	Fluorescence RPA			Semi-nested PCR		
	+	−	Total	+	−	Total
+	30	0	30	29	1	30
−	0	11	11	0	11	11
Total	30	11	41	29	12	41

Taken together, Kato-Katz identified 30 samples as *N. americanus* positive and 11 samples as negative. In the semi-nested PCR analysis, a target band of 250 bp was identified in 29 samples, which were confirmed to be homologous to the *N. americanus* through Sanger sequencing and comparison with the NCBI database. Twelve samples showing no target band were classified as negative (S2 Fig). In fluorescence RPA assay, 30 samples exhibited a robust beta-fluorescence curve within 20 minutes (Fig 6). The remaining 4 samples showed fluorescence amplification, but they did not follow the ‘S’-shaped trend amplification curve [24] of fluorescent RPA as the positive control, thus classified as negative [17]. The other 7 samples did not exhibit fluorescence amplification and were therefore classified as negative. Based on these data, the sensitivity and specificity of semi-nested PCR compared to Kato-Katz were 96.7% (29/30) and 100% (11/11), respectively. Similarly, the sensitivity and specificity of fluorescent RPA compared to Kato-Katz were both 100% (30/30 for sensitivity and 11/11 for specificity) (Table 5). And the fluorescent RPA demonstrated 100% (29/29) sensitivity and 91.7% (11/12) specificity when compared to the semi-nested PCR.

### 3.3. Detection of the fecal samples collected from field sites

**Fig 7:**
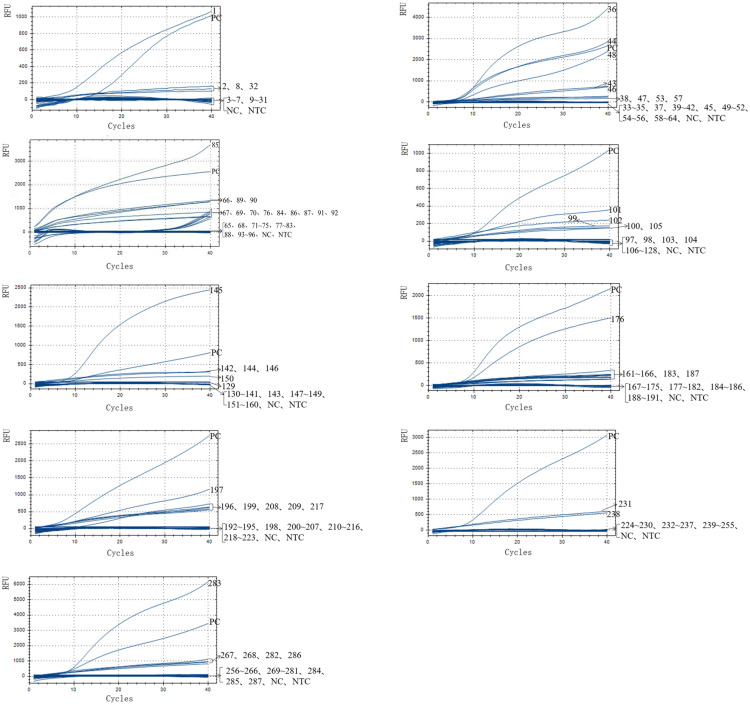
Detection results of *N. americanus* in 287 human fecal samples collected from the field via Fluorescent RPA (Line 1-287: Human fecal samples from the field; PC: Positive control; NC: Negative control; NTC: No template control).

In the analysis of the 287 samples, 40 were identified as positive for *N. americanus* using the Kato-Katz, 58 tested positive for *N. americanus* through fluorescent RPA (Fig 7), and 34 samples were confirmed positive for *N. americanus* via semi-nested PCR (S3 Fig). PCR testing for *A. duodenale* was performed on 62 samples that tested positive for *N. americanus*, and all results were negative for *A. duodenale* (S4 Fig).

**Fig 8:**
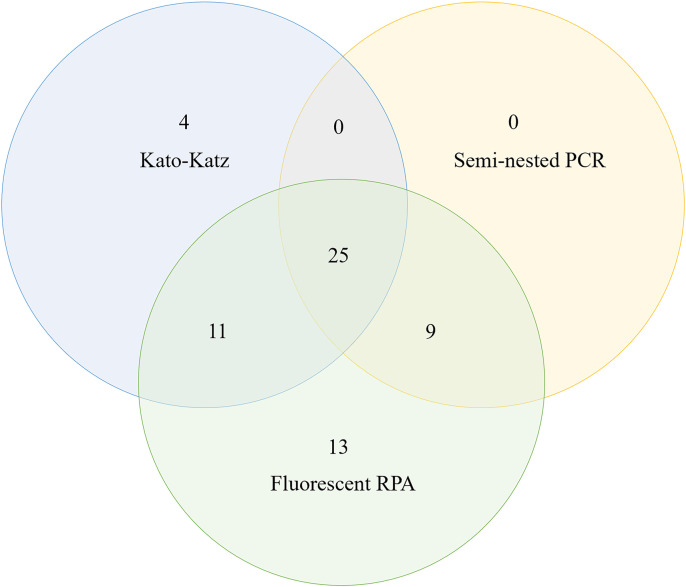
Venn diagram of the positive results of the three assays.

Among the 62 samples that tested positive for *N. americanus*, 25 samples were positive by all three methods, 11 were positive by both Kato-Katz and fluorescent RPA, 9 were positive by both fluorescent RPA and semi-nested PCR, 13 were positive by fluorescent RPA alone, and 4 were positive by Kato-Katz alone, as shown in Fig 8. Additionally, 4 samples (No. 2, 10, 25, 26) were detected exclusively by the Kato-Katz, with egg counts of 1.5, 0.5,1 and 2, respectively, as shown in Table 6.

**Table 6:** Number of eggs in the 40 positive fecal samples detected by the Kato-Katz and the detection results of semi-nested PCR and fluorescent RPA on these samples.

Number	Kato-Katz (average egg number)	Semi-nested PCR	Fluorescence RPA
1	27	+	+
2	1.5	−	−
3	2.5	+	+
4	3.5	+	+
5	3	+	+
6	2	+	+
7	41	+	+
8	81	+	+
9	8	+	+
10	0.5	−	−
11	3	+	+
12	8	+	+
13	485	+	+
14	40	+	+
15	480	+	+
16	7	+	+
17	43	+	+
18	4	+	+
19	2	+	+
20	2	−	+
21	8	+	+
22	8	+	+
23	5	−	+
24	1	−	+
25	1	−	−
26	2	−	−
27	1	−	+
28	5	−	+
29	1	−	+
30	9	+	+
31	5	+	+
32	1	−	+
33	1	−	+
34	1	−	+
35	3	−	+
36	4	+	+
37	7	+	+
38	2	−	+
39	5	+	+
40	6	+	+

The specificity and sensitivity of the three methods exhibit differences when compared to one another (show in Tables 7–9). Out of 287 samples examined by the Kato-Katz, 13.94% (40/287) tested positive for *N. americanus*. These samples underwent subsequent testing with fluorescent RPA and semi-nested PCR assays. The results indicated that 20.21% (58/287) samples tested positive for *N. americanus* via fluorescent RPA. Additionally, 11.85% (34/287) samples were identified as positive through semi-nested PCR. And the sensitivity of the fluorescent RPA method compared to the semi-nested PCR method was 100% (34/34), while the specificity was 90.5% (229/252).

**Table 7:** Comparison of the Kato-Katz with semi-nested PCR for detecting human fecal samples.

Kato-Katz	Semi-nested PCR		Total
	+	−	
+	25	15	40
−	9	238	247
Total	34	253	287

**Table 8:** Comparison of the Kato-Katz with fluorescent RPA for detecting human fecal samples.

Kato-Katz	Fluorescence RPA		Total
	+	−	
+	36	4	40
−	22	225	247
Total	58	229	287

**Table 9:** Comparison of the fluorescent RPA with Semi-nested PCR for detecting human fecal.

Fluorescence RPA	Semi-nested PCR		Total
	+	−	
+	34	24	58
−	0	229	229
Total	34	253	287

## 4. Discussion

Despite being classified as a neglected tropical disease, the economic and health burden of hookworm exceeds published estimates for several diseases that have received comparatively more attention, such as rotavirus. Moreover, some large countries transitioning to higher income statuses continue to grapple with significant hookworm burdens [2,30]. RPA operates effectively within a temperature range of 37–42°C, capable of detecting target DNA even at concentrations as low as 1–10 copies and providing results in under 20 minutes [31–34]. In this study, we successfully developed a fluorescent RPA assay for the detection of *N. americanus*, based on the *ITS2* gene to generate an ‘S’-shaped trend amplification curve as the criterion for a positive result. The analysis included PC, NTC, and NC, with negative samples showing trends similar to those of the NC and NTC, demonstrating high sensitivity and specificity. This assay offers significant advantages in addressing the current *N. americanus* epidemic and markedly improves the efficiency of detecting *N. americanus* eggs in fecal samples. The effective diagnostic sensitivity of this test is demonstrated at 1 fg/µL.

Worm egg detection through fecal egg counting techniques represents a pivotal methodology for assessing parasite burden, resistance profiles, and diagnosing diseases across diverse animal species [35,36]. In this study, we found no statistically significant difference in the detection rate between semi-nested PCR and fluorescent RPA for fecal samples containing three eggs (P>0.05). However, fluorescent RPA demonstrated higher detection efficacy than semi-nested PCR for fecal samples containing a single egg (P=0.02<0.05). These results suggest that while semi-nested PCR is effective for detecting samples with low levels of infection (EPG=15 in this study) [37], fluorescent RPA shows superior sensitivity at very low levels of infection (EPG=5 in this study). Over the past 15 years, the rapid development of RPA has been driven by its short response time and high sensitivity [38]. As noted by Frimpong et al. [39] and Lee et al. [33]. Compared to PCR, RPA enables isothermal amplification at 37°C to 42°C, which reduces reaction times and simplifies equipment requirements, making it more suitable for field applications. Current molecular diagnostic techniques for *N. americanus* include semi-nested PCR, qPCR, and others [12,14,25,28,29].

In this study, although both fluorescent RPA and qPCR are based on fluorescent molecular detection methods and they are more directly comparable compared to semi-nested PCR, we selected semi-nested PCR due to its sufficient sensitivity to meet the requirements of the study [40–43]. This choice was made based on its established reliability and extensive validation, as well as its demonstrated effectiveness in detecting positives in the 3-egg experiment. Furthermore, after conducting a comprehensive literature review, we found that the semi-nested PCR method has consistently shown effective performance in numerous studies, making it a reliable benchmark for our work [28,29,44].

When different methods were used to detect the 287 human fecal samples collected from different field sites, with Kato-Katz as the benchmark, the sensitivity rates of fluorescent RPA and semi-nested PCR were 90.0% (95% *CI*: 86.5% to 93.5%) and 62.5%, respectively, while the specificity of fluorescent RPA and semi-nested PCR stood at 91.1% (95% *CI*: 87.8% to 94.4%) and 96.4%. The positive predictive value (PPV) of the fluorescence RPA assay was 62.1% (95% *CI*: 48.4% to 74.5%), and the negative predictive value (NPV) was 98.3% (95% *CI*: 95.6% to 99.5%). The sensitivity of the fluorescent RPA method compared to the semi-nested PCR method was 100% (34/34, 95% *CI*:100%), while the specificity was 90.5% (229/252, 95% *CI*:87.1% to 93.9%). The discrepancy between Kato-Katz negative results and semi-nested PCR and fluorescent RPA negative results, as well as between Kato-Katz positive results and semi-nested PCR and fluorescent RPA positive results could be due to factors such as the lower sensitivity of the Kato-Katz, failure to detect eggs promptly after slide preparation, or egg degradation [45,46]. We observed that out of 287 samples, of 58 positive samples detected by fluorescent RPA, 13 samples were tested positive exclusively through fluorescent RPA, possibly due to the fact that RPA has a higher limit of detection than semi-nested PCR [28].

Among the 273 samples from Hainan, 46 were found to be positive using the fluorescent RPA. Of these, 13 were positive in both the Kato-Katz and semi-nested PCR, 11 were positive by Kato-Katz, and 9 were positive by semi-nested PCR, indicating a higher sensitivity of the molecular detection methods[25,32,36,37]. The results of China’s National Soilborne Nematode Infection Monitoring in 2019 showed that the hookworm infection rate in Hainan Province was 2.68% [47]. However, the infection rate of *N. americanus* in this study was higher than this value, suggesting that the actual infection rate of *N. americanus* in Hainan Province may have been previously underestimated. Nonetheless, molecular detection methods also have some limitations, which can arise from various factors. For example, in the four samples (No. 2, 10, 25, 26) tested with the Kato-Katz method, the egg counts were 1.5, 1, 2, and 0.5 respectively, while the molecular test results were negative. Although fecal samples were homogenized before DNA extraction, this discrepancy suggests a potential sampling error, possibly resulting from very low infection levels and the non-homogeneous distribution of eggs in the feces, which may have led to the selection of samples containing no eggs for DNA extraction [48–50]. Nevertheless, out of a total of 287 samples, the molecular assay identified 22 samples as positive, whereas the Kato-Katz returned negative results for these samples, suggesting that the molecular assay generally exhibits higher sensitivity than the Kato-Katz [19,36,37,51].

The fluorescent RPA assay developed in this study can deliver timely results, enhancing its feasibility for real-world applications, particularly in resource-limited settings where rapid diagnostics are crucial. The capability of RPA to detect target DNA at very low concentrations makes it an invaluable tool for early detection and intervention, which are essential for controlling the spread of *N. americanus* and mitigating its impact on public health. The adoption of this fluorescence RPA in diagnostic protocols can improve the accuracy of hookworm infection surveillance and contribute to more effective disease management and control strategies, particularly in endemic regions. Additionally, the T8 Isothermal Diagnostics Instrument (Axxin, Victoria, Australia) can be utilized to visualize the final results of fluorescent RPA in field settings. Therefore, it holds significant potential as a detection tool in public health for primary care and screening, enhancing the efficiency and accuracy of *N. americanus* detection. In scientific research, it can serve as a valuable tool for studying the epidemiological characteristics of *N. americanus* disease and evaluating drug efficacy.

Numerous constraints were identified due to the investigative nature of this study. Firstly, variations in molecular assay results may be attributed to differences in sampling hotspot, underscoring the necessity for continued vigilance to ensure the reliability and consistency of molecular analyses. Secondly, the methods of DNA extraction are of paramount importance in any molecular method, as they directly impact the performance of RPA and its field application. Simplifying DNA extraction procedures and ensuring their quality present significant challenges that require attention.

## 5. Conclusion

In summary, the rapid detection method for *N. americanus* based on fluorescent RPA technology established in this study offers significant advantages and shows broad application prospects. It is characterized by high sensitivity, high specificity, and a rapid operation procedure, making it highly practical for large-scale detection and use in primary healthcare institutions. This method has wide application potential and great development prospects in the field of *N. americanus* disease control and research.

## Supporting information

S1 Fig (a) Detection results of fecal samples containing 1 egg via semi-nested PCR. (Number 1–20: human fecal samples containing 1egg; PC: positive control; NC: negative control;) (b) Detection results of fecal samples containing 3 eggs via semi-nested PCR (Number 1–20: human fecal samples containing 3 eggs; PC: Positive control; NC: Negative control;).
(DOCX)

S2 Fig Detection results of *N. americanus* in 41 human fecal samples via semi-nested PCR for sensitivity and specificity assessment (Number 1–41: human fecal samples; PC: Positive control; NC: Negative control;). (DOCX)

S3 Fig Detection results of *N. americanus* in 287 human fecal samples collected from the field via semi-nested PCR (Number 1–287: human fecal samples; PC: Positive control; NC: Negative control;). (DOCX)

S4 Fig PCR testing for *A. duodenale* was performed on 62 samples that tested positive for *N. americanus* (M: Marker; P: Positive control; NC: Negative control; Number: 62 samples). (DOCX)

S1 Data Experimental data for Figs 2–7. (XLSX)
