# Supplementary material for: Development and evaluation of fluorescent recombinase polymerase amplification (RPA)-based method for rapid detection of Necator americanus
Source: PLoS Negl Trop Dis. 2025 Apr 8;19(4):e0013007. doi: 10.1371/journal.pntd.0013007 (PMC12011292; doi:10.1371/journal.pntd.0013007)
Supplement: S1 Fig — (Number 1–20: human fecal samples containing 1egg; PC: positive control; NC: negative control;) (b) Detection results of fecal samples containing 3 eggs via semi-nested PCR (Number 1–20: human fecal samples containing 3 eggs; PC: Positive control; NC: Negative control;). (DOCX) [file pntd.0013007.s001.docx]

**Supplementary 1 Fig. Detection of fecal samples containing varying quantities of *N.***

***americanus* eggs via semi-nested PCR**


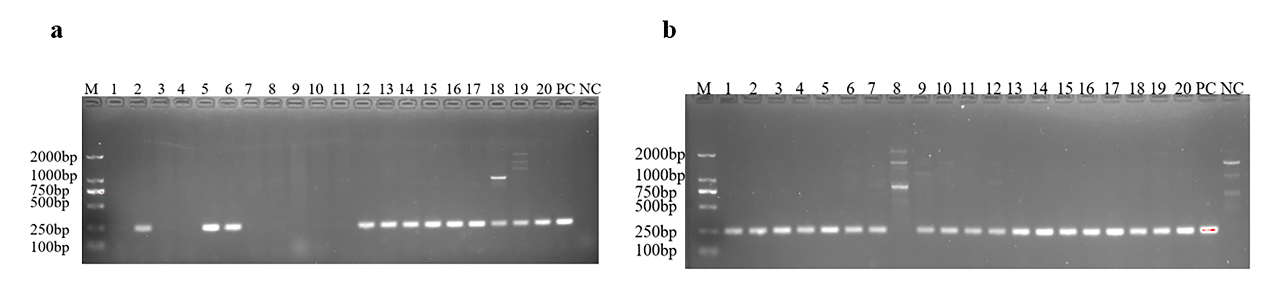


S1 Figure. (a) Detection results of fecal samples containing 1 egg via semi-nested PCR. (Number 1-20: human fecal samples containing 1egg; PC: positive control; NC: negative control;) (b) Detection results of fecal samples containing 3 eggs via semi-nested PCR (Number 1-20: human fecal samples containing 3 eggs; PC: Positive control; NC: Negative control;)
