# Supplementary material for: Development and evaluation of fluorescent recombinase polymerase amplification (RPA)-based method for rapid detection of Necator americanus
Source: PLoS Negl Trop Dis. 2025 Apr 8;19(4):e0013007. doi: 10.1371/journal.pntd.0013007 (PMC12011292; doi:10.1371/journal.pntd.0013007)
Supplement: S2 Fig — (DOCX) [file pntd.0013007.s002.docx]

**Supplementary 2 Fig.** Detection results of *N. americanus* in 41 human fecal samples via

semi-nested PCR


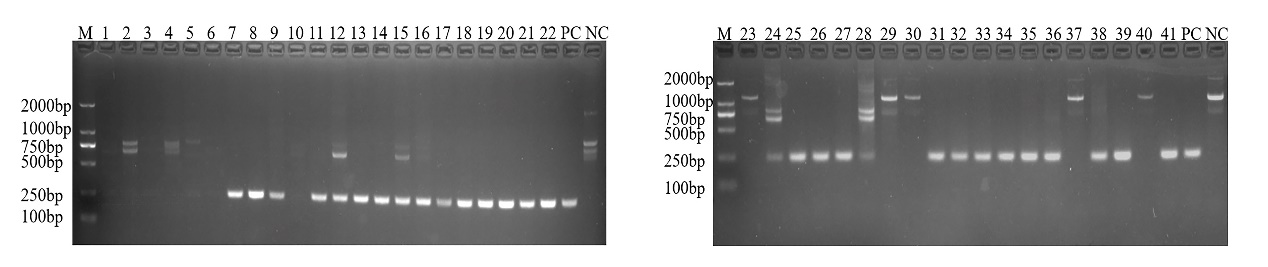


S2 Figure. Detection results of *N. americanus* in 41 human fecal samples via semi-nested PCR

for sensitivity and specificity assessment (Number 1-41: human fecal samples; PC: Positive

control; NC: Negative control;)
