# Supplementary material for: Development and evaluation of fluorescent recombinase polymerase amplification (RPA)-based method for rapid detection of Necator americanus
Source: PLoS Negl Trop Dis. 2025 Apr 8;19(4):e0013007. doi: 10.1371/journal.pntd.0013007 (PMC12011292; doi:10.1371/journal.pntd.0013007)
Supplement: S3 Fig — (DOCX) [file pntd.0013007.s003.docx]

**Supplementary 3 Fig.** Detection results of *N. americanus* in 287 human fecal samples collected

from the field via semi-nested PCR


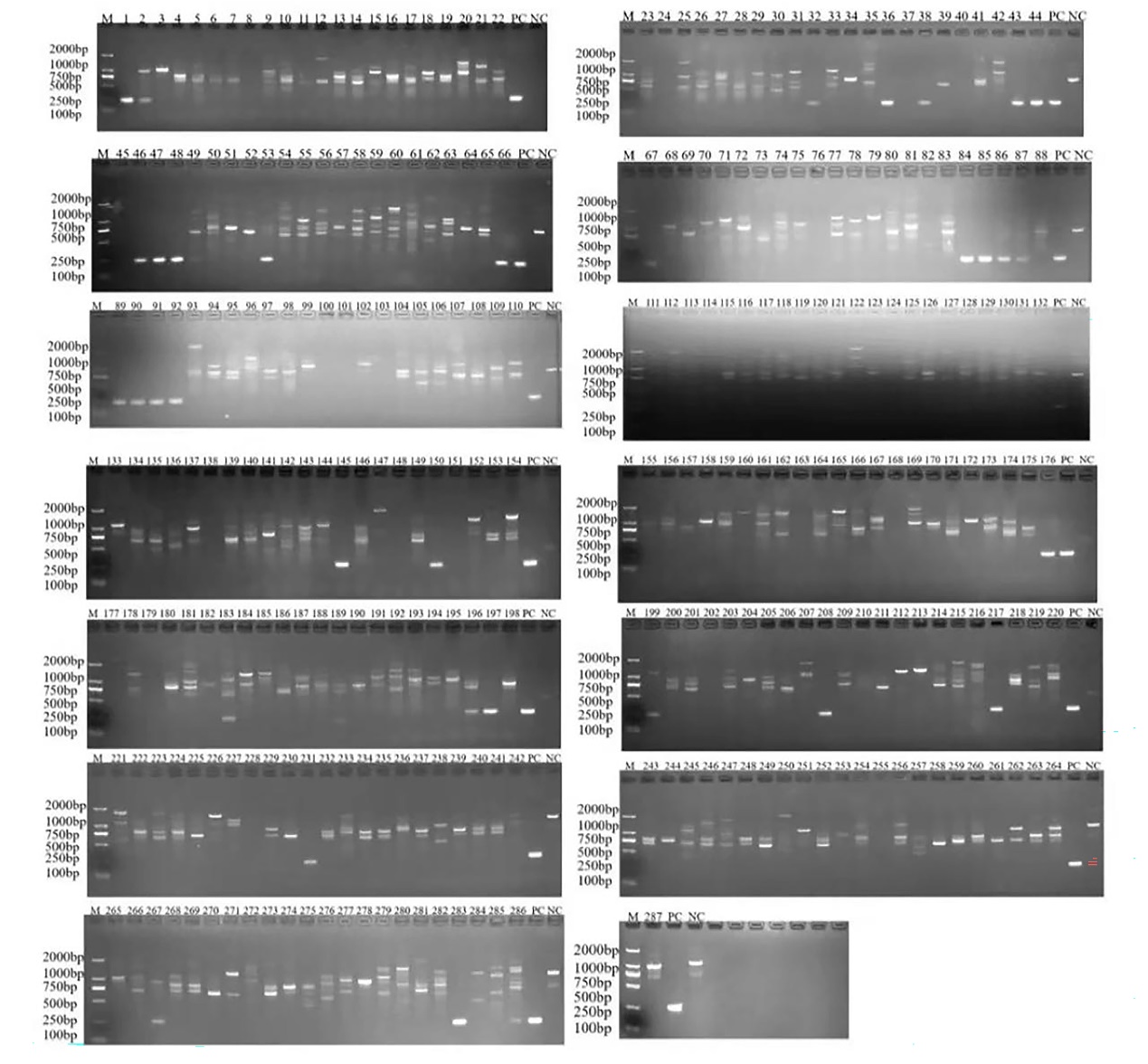


S3 Figure. Detection results of *N. americanus* in 287 human fecal samples collected from the field via semi-nested PCR (Number 1-287: human fecal samples; PC: Positive control; NC: Negative control;)
