# Supplementary material for: Development and evaluation of fluorescent recombinase polymerase amplification (RPA)-based method for rapid detection of Necator americanus
Source: PLoS Negl Trop Dis. 2025 Apr 8;19(4):e0013007. doi: 10.1371/journal.pntd.0013007 (PMC12011292; doi:10.1371/journal.pntd.0013007)
Supplement: S4 Fig — (DOCX) [file pntd.0013007.s004.docx]

**Supplementary 4 Fig.** PCR testing for *A. duodenale* was performed on 62 samples that

tested positive for *N. americanus*


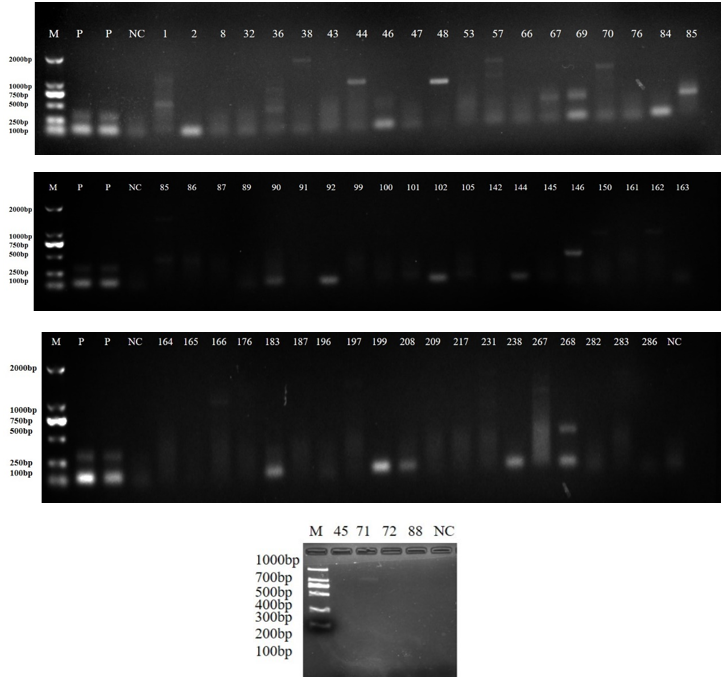


S4 Figure. PCR testing for *A. duodenale* was performed on 62 samples that tested positive for *N. americanus* (M: Marker; P: Positive control; NC: Negative control; Number: 62 samples)
